# Effects of Auditory Frequency Stimulation on Balance and Rehabilitation Outcomes in Patients With Stroke: A Randomized Case‐Control Study

**DOI:** 10.1002/brb3.70671

**Published:** 2025-07-07

**Authors:** Ruijin Chen, Lihua Jin, Jin Song, Juchuan Dong, Yongmei Li

**Affiliations:** ^1^ Department of Rehabilitation Therapy Second Affiliated Hospital of Kunming Medical University Kunming Yunnan China; ^2^ Department of Rehabilitation Medicine Kunming Medical University Kunming Yunnan China

**Keywords:** auditory stimulation, balance, sensory stimulation, stroke

## Abstract

**Background:**

Although the role of sensory input in maintaining balance is well established, the effects of various auditory frequency stimulations, such as binaural beat stimulation (BBS), on patients with central nervous system injuries, particularly those who have experienced a stroke, remain inadequately understood.

**Methods:**

This randomized case‐control study compared the functional outcomes between patients with stroke undergoing BBS during rehabilitation training and those receiving traditional music therapies. The assessed primary outcomes included balance function, balance confidence, lower‐limb function, and activities of daily living, cognitive abilities, and depression scores. Subsequently, correlations between outcome parameters were analyzed.

**Results:**

A total of 27 patients with stroke completed the study protocol (BBS *n* = 15, control *n* = 12). Post‐intervention analyses revealed improvements in balance‐related indicators, particularly the scores of the Berg Balance Scale (BBSc) and Mini‐Balance Evaluation Systems Test (Mini‐BESTest), across both groups. However, the Barthel index (BI) and Beck depression inventory scores (BDIS) in the BBS group exhibited significantly greater improvements than those in the control group. Correlation analyses indicated that changes in balance‐related scores within the BBS group were associated with improvements in lower‐limb function, mood, and activities of daily living. Conversely, the control group observed correlations among the internal components of the balance‐related scales.

**Conclusion:**

Appropriate BBS stimulation appears to enhance balance, likely owing to the synergistic effects of music and rhythm. Further research focusing on brain function is necessary to elucidate the mechanisms underlying the therapeutic efficacy of BBS interventions.

## Graphical abstract

A novel opportunity for intervention in post‐stroke balance disorders has been identified, as we have discovered that the binaural beat stimulation (BBS) may significantly enhance patients' balance function and subsequently improve activities of daily living more effectively than conventional rehabilitation methods.

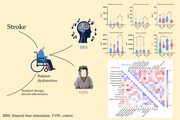

## 1 Introduction

Balance is integral to daily functioning, and any balance impairment in adults can significantly affect postural stability, thereby increasing the incidence of falls (Serrador et al. 2009). Falls are particularly prevalent among older adults, are even more frequent in patients with stroke, and are often linked to gait abnormalities, lower limb dysfunction, and balance deficits. Thus, falls are one of the most common post‐stroke complications (McLean 2004; Nonnekes et al. 2019). Postural balance during standing is maintained through the continuous coordinated engagement of the musculoskeletal, visual, proprioceptive, and vestibular systems (Lafond et al. 2004). In contrast, during dynamic activities, posture control is more reliant on the cognitive task load and the sustained integration of multisensory inputs (Takakusaki 2017).

The vestibular and auditory systems are considerably interconnected, particularly considering the integral roles of the inner ear vestibular organs and the Corti sensory system. Anatomical proximity and physiological connections suggest a potential interaction between the auditory and vestibular organs. Consequently, auditory stimuli are hypothesized to influence the postural stability of the human body (Mainenti et al. 2007; Park et al. 2011). Recently, an increasing number of studies have investigated the effects of auditory information on balance regulation (Seiwerth 2023). Some researchers have even proposed that the auditory system should be considered one of the four principal components of postural regulation alongside the vestibular, visual, and proprioceptive systems (Dlugaiczyk 2020). Despite an increasing number of research groups investigating this issue in recent years, evidence for the influence of auditory stimuli on postural regulation is limited.

Błażkiewicz et al. (2024) observed that sensory stimulation, particularly in the form of irritating sounds, can exacerbate irregularities in postural control, particularly in conditions where visual feedback is absent. Similarly, Belluscio et al. (2023) demonstrated that auditory stimulation can activate the prefrontal cortex (PFC) and other cortical regions implicated in postural control, with varying frequencies eliciting distinct activation patterns. These findings suggest that specific sound frequencies are critical for the recruitment of cognitive resources and the regulation of postural control. Furthermore, specific auditory stimuli may significantly modulate the relationship between cortical activity and postural control, particularly in populations with neurological impairment. Sounds can serve as interference factors, leading to alterations in sensory processing and attentional focus, thereby affecting the mechanisms of postural stability. Evidence suggests that low‐frequency sounds, as opposed to silence, can reduce postural sway (Park et al. 2011; Easton et al. 1998). This implies that certain auditory stimuli may be beneficial for patients with postural instability. However, research in this domain remains nascent and warrants further investigation.

Binaural beat stimulation (BBS) is a phenomenon characterized by the presentation of auditory stimuli at different frequencies in each ear, resulting in the perception of sine waves at a specific frequency within the brain (Lee et al. 2022). Research suggests that BBS is primarily processed in the medial superior olive nucleus of the brainstem rather than within the central auditory neurons of the auditory nerve (Draganova et al. 2008). Nonetheless, the precise mechanisms underlying this phenomenon remain ambiguous, as BBS is not solely comprised of beats but is often accompanied by musical rhythms (Frick and Young 2019).

Initially, we used BBS interventions to enhance patients’ attention during rehabilitation exercises in acoustically challenging treatment environments. However, emerging evidence in our clinical work suggests that BBS may provide additional benefits. Accordingly, we hypothesize that the implementation of the BBS intervention may confer benefits to stroke patients by enhancing balance. This improvement is potentially mediated through the intervention's effects on other cognitive functions, such as attention.

## 2 Methods

### 2.1 Study Design

This randomized controlled trial was conducted from August 2023 to April 2024 at the biggest rehabilitation center in Yunnan province in Southwest China. Ethical approval was obtained from the Tertiary Hospital Ethics Committee (no. PJ‐2023‐189). The trial is registered with the Chinese Clinical Trial Registry (no. ChiCTR2400088967). Prior to participation, all individuals received comprehensive information regarding the study objectives and procedures, and written informed consent was obtained from each patient. Random assignment was conducted with the random number table approach. In total, 30 individuals were enrolled and assigned to either the BBS group (*n* = 15) or the control (CON) group (*n* = 15).

### 2.2 Participants

This study included individuals aged 20–80 years who had a confirmed diagnosis of hemorrhagic or ischemic stroke and whose disease course was less than 1 year. Additionally, the participants were required to have no history of hearing impairment or related diseases, a Mini‐Mental State Examination (MMSE) score of 24 or higher, and a Berg Balance Scale (BBSc) score of 20–40 points. As the intervention relied on auditory stimulation, patients with a history of audiogenic seizures or other psychiatric, audiovestibular, or systemic disorders were excluded.

### 2.3 Intervention

The intervention for the BBS group consisted of a BBS program (therapeutic listening) that involved the use of modified music delivered through a headset during occupational therapy sessions. This intervention was administered once daily over 14 days. Throughout the intervention, the participants continued to receive standard rehabilitation therapies, including physical therapy, physiotherapy, and other relevant interventions, but no additional occupational therapy. The CON group received the same rehabilitation regimen as the BBS group; however, the BBS program was replaced with unmodified standard music. The BBS group used and repeatedly played a single track from the “Gravity Grapes” album in the Quickshifts series (https://vitalsounds.com/product/quickshift‐gravitational‐grape/). When healthy individuals (non‐participating staff members) were exposed to music modified by the BBS‐modified music, they reported an enhanced perception of rhythm. The volume was adjusted according to the patients' comfort level to ensure that it was neither too distracting nor too quiet to be heard.

### 2.4 Outcome Measures

Balance, lower‐limb, and other functions were measured before baseline assessment (T0) and after intervention assessment (T1) 14 days of intervention. The observer who performed the evaluation was blind to the group.

#### 2.4.1 Balance Function

Balance function was assessed using the HUBER 360 platform, Mini‐Balance Evaluation Systems Test (Mini‐BESTest), and BBSc. The program on the HUBER platform includes a stability test (which measures the center of gravity in patients with both eyes either closed or open to assess the balance function), a unipodal test (balance on the left or right leg), limits of stability, a walking step, and a mobility restriction test (assesses the patient's mobility limitations and ability to work) (Mureșan et al. 2022). In patient populations with stroke, both the Mini‐BESTest and BBSc have good inter‐ and intra‐rater reliability (Barzideh et al. 2020). The activity‐specific balance confidence (ABC) questionnaire was used to assess participants’ self‐confidence during balance‐related tasks. The participants were asked to rate their confidence while performing such tasks.

#### 2.4.2 Lower‐limb Function

The Fugl–Meyer assessment (FMA) and timed up and go (TUG) test were used to assess lower‐limb function. The FMA is a reliable, valid, and responsive measure of motor function after stroke (Gladstone et al. 2002). The TUG test is an objective assessment of lower‐limb function and locomotion.

#### 2.4.3 Activities of Daily Living (ADLs) and Assessment of Cognition and Depression

The Barthel Index (BI), MMSE, and Beck Depression Inventory (BDI) were used to assess ADLs, cognition, and depressive symptoms, respectively. Abnormal balance may result in reduced ADLs (Zhang et al. 2021). Moreover, balance function is negatively related to other functions, such as cognitive impairment and depression (Lee et al. 2022); therefore, these factors were included in our study.

### 2.5 Statistical Analysis

SPSS 26.0 software (IBM, NY, USA) was used for statistical analyses. We compared baseline demographic characteristics and post‐intervention changes between the two groups. Based on whether data conformed to the normal distribution, continuous variables are expressed as the mean ± standard deviation or median (min‐max). Categorical variables are expressed as frequencies and percentages. To evaluate differences between the two groups, the independent sample *t*‐test or Mann–Whitney *U* test was used for continuous variables and the chi‐squared or Fisher's exact test for categorical data. To assess differences within each group, the paired sample *t*‐test or Wilcoxon test was used. To determine changes in functional improvement to clarify whether the patient's functional improvement is due to the intervention alone or due to concomitant improvement in other functions, the delta value (owing to some functions that may have decreased than baseline, the delta value (T1–T0 value) is not expressed as an absolute value in the present study) was calculated to compare correlations, and Spearman's correlation test was used for bivariate correlation analysis in each group. Statistical significance was set at *p* < 0.05. Data were plotted using the GraphPad Prism 8.0 program (GraphPad Inc., San Diego, CA, USA).

## 3 Results

### 3.1 Demographic and Clinical Characteristics at Baseline

Of the 30 enrolled participants, three in the CON group dropped out of the trial for personal reasons or changes in their condition. Thus, 27 participants completed the study. Their average age was 58 ± 11.29 years, and the duration since disease onset ranged from 10 to 330 days. At T0, balance function, self‐confidence in balance, lower‐limb function, cognition, and depressive symptoms were not significantly different between the two groups (Table 1). No safety issues or adverse events were reported during the trial in either group.

**TABLE 1:** Demographic characteristics of each study group.

		BBS group	Con group	*p* value
Gender	Male	12	6	0.107
	Female	3	6	
Age (year)		55.13 ± 10.33	61.5 ± 11.87	0.149
Stroke onset (days)		93.0 ± 93.94	81.08 ± 120.36	0.549
Type of stroke	Hemorrhage	8	3	0.144
	Ischemic	7	9	
Stability‐area (eyes open) (mm^2^)		782.4 (257.9, 11443.1)	528.6(107.8, 5394.5)	0.306
Stability‐area (eyes closed) (mm^2^)		1928 (800.3, 9539.6)	1061.4 (442.6, 4705.3)	0.262
Stability‐speed (eyes open) (mm/s)		27.1 (11.1, 216)	23.65 (13.1,52.5)	0.143
Stability‐speed (eyes closed) (mm/s)		34.4 (17.1, 87.9)	34.85 (15.5,97.3)	0.407
Unipodal left length (mm^2^)		2495.8 (0, 37903.1)	8165.55 (0,69232.3)	0.493
Unipodal right length (mm^2^)		11087.1 (0, 69369.3)	5975.15 (0,38397.1)	0.713
Limits of stability (mm^2^)		17039.5 (849.6, 44840.5)	21200.1 (12011.3, 155661.4)	0.223
Walking test (step)		50.6 ± 24.8	50.08 ± 25.96	0.958
Mobility restriction test (°)		32.07 ± 23.4	40.83 ± 10.42	0.240
Mini_BESTest		17 (9, 19)	16 (10, 24)	0.731
BBSc		35 (20, 40)	35 (24, 41)	0.713
ABC		78.4 ± 22.43	74.92 ± 37.61	0.767
FMA		33 ± 8.59	39.25 ± 13.46	0.155
TUG		44 (35, 72)	44 (29, 76)	0.591
BI		64.6 ± 10.99	58.58 ± 11.19	0.173
MMSE		29 (28, 30)	28 (28, 30)	0.505
BDI		5 (0, 26)	6 (2, 13)	0.573

**Abbreviations**: ABC, activity‐specific balance confidence questionnaire; BBSc, Berg Balance Scale; BDI, Beck Depression Inventory; BI, Barthel index; FMA, Fugl–Meyer assessment; Mini‐BESTest, Mini‐Balance Evaluation Systems Test; MMSE, Mini‐Mental State Examination; TUG, timed up and go test.

### 3.2 Balance Function

**FIGURE 1:**
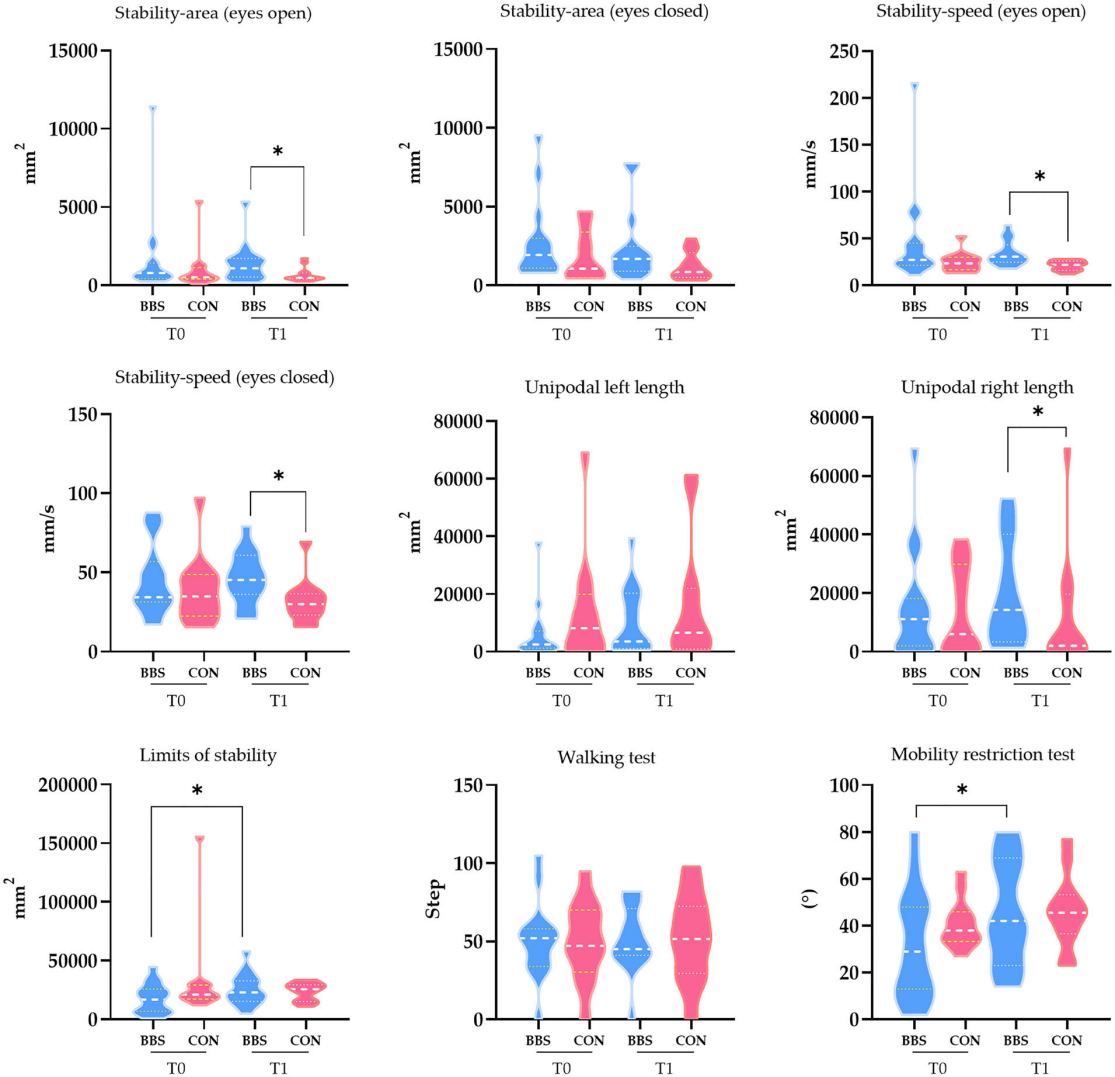
Change in balance function in the two study groups. **Abbreviations**: BBS, binaural beat stimulation; CON, control; T0, baseline assessment; T1, after intervention assessment.

After the intervention, both stability area, stability speed during eye opening, stability speed with eyes closed, and unipodal right length were significantly different between the two study groups (*p* = 0.036, 0.002, 0.017, and 0.040, respectively). The limits of the stability test (*p* = 0.041) and mobility restriction test (*p* = 0.004) changed significantly only in the BBS group after the intervention (Figure 1). In both groups, the Mini‐BESTest and BBSc scores after the intervention showed significant changes compared with those at baseline (*p* = 0.001 and *p* = 0.002, respectively); however, no significant difference was observed between the two groups at T1 (Table 2). Similar changes were observed after the intervention when comparing the ABC scores within and between groups; BBS and CON groups (both *p* < 0.001) showed significant score changes compared to baseline, but no difference between the two groups was found at T1 (*p* = 0.932; Table 2).

**TABLE 2:** Post‐intervention changes in functional scales in the two study groups.

	BBS group			Con group	*p* value
	T0	T1	T0	T1	
Mini‐BESTest	17 (9, 19)	24 (15, 324)^†^	16 (10, 24)	21 (14, 27)^†^	0.148
BBSc	35 (20, 40)	45 (30, 54)^†^	35 (24, 40)	41.5 (38, 52)^†^	0.433
ABC	78.4 ± 22.43	92 ± 17.23^†^	74.92 ± 37.61	92.92 ± 36.43^†^	0.932
FMA	33 ± 8.59	45.33 ± 12.62^†^	39.25 ± 13.46	44.83 ± 12.88^†^	0.920
TUG	44 (35, 72)	35 (30, 58)^†^	44 (29, 76)	34 (25, 68)^†^	0.922
BI	64.6 ± 10.99	76.87 ± 7.02^†^	58.58 ± 11.19	68.92 ± 10^†^	**0.023** ^*^
MMSE	29 (28, 30)	29 (28, 30)	28 (28, 30)	28 (28, 30)	0.505
BDI	5 (0, 26)	3 (0, 23)^†^	6 (2, 13)	4.5 (1, 18)	0.257

**Abbreviations**: ABC, activity‐specific balance confidence questionnaire; BBSc, Berg Balance Scale; BBS, binaural beat stimulation; BDI, Beck Depression Inventory; BI, Barthel index; CON, Control; FMA Fugl–Meyer assessment; Mini‐BESTest, mini‐balance evaluation systems test; MMSE, Mini‐Mental State Examination; T0, baseline assessment; T1, after intervention assessment; TUG, timed up and go test.

* *p*‐value calculated between the two groups at T1 using the independent sample t‐test.

^†^ *p*‐value calculated with‐in the group (T0 vs. T1) using the paired sample t‐test.

### 3.3 Lower‐limb Function

In both BBS and CON groups, we found significant within‐group differences in the FMA (*p* < 0.001 and *p* = 0.005, respectively) and TUG test (*p* = 0.001 and *p* = 0.016, respectively). After the intervention, neither the FMA nor TUG test results significantly differed between the groups (Table 2).

### 3.4 Assessment of ADLs, Cognition, and Depression

The BIs in the BBS and CON groups (both *p* < 0.001) revealed significant improvements after the intervention; moreover, the BBS group showed at T1 a greater improvement than the CON group (*p* = 0.023). No change was found in either group when comparing the MMSE scores before and after the intervention. Only the BDI score in the BBS group showed a significant change after the intervention (*p* = 0.002; Table 2).

### 3.5 Correlations of Functional Scores Between the Two Study Groups

In the BBS group, the delta value of FMA was moderately positively correlated with the delta values of the BI (*r* = 0.684, *p* = 0.005) and ABC score (*r* = 0.528, *p* = 0.043) but moderately negatively correlated with the delta value of the stability area (eyes closed *r* = −0.523, *p* = 0.046). Moreover, changes in mobility restriction scores were moderately positively correlated with BBSc score changes (*r* = 0.524, *p* = 0.045) in this group. The BDI showed a moderate negative correlation with stability speed (eyes closed *r* = −0.574, *p* = 0.025), and the BI showed a moderate negative correlation with the TUG value (*r* = −0.536, *p* = 0.039) in the BBS group.

**FIGURE 2:**
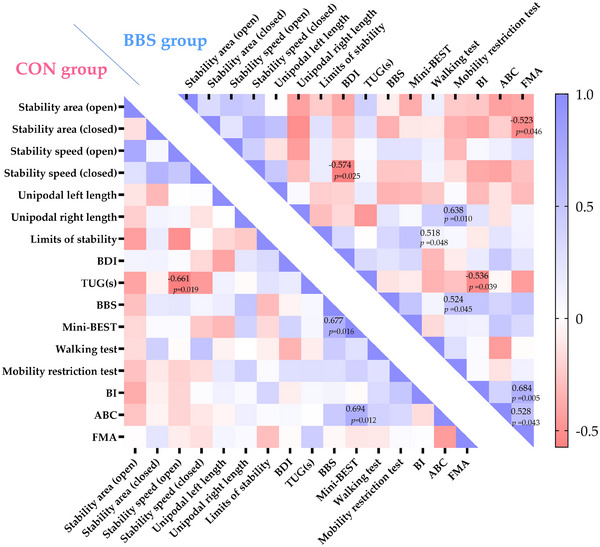
**Correlation heatmap of the two study groups. Abbreviations**: ABC, activity‐specific balance confidence questionnaire; BBSc, Berg Balance Scale; BDI, Beck Depression Inventory; BI, Barthel index; FMA, Fugl–Meyer assessment; Mini‐BESTest, Mini‐Balance Evaluation Systems Test; TUG, timed up and go test. *p‐value calculated using Spearman's correlation test.

In the CON group, the change in the Mini‐BESTest score showed a moderate positive correlation with the delta values of the ABC scale (*r* = 0.694, *p* = 0.012) and BBSc (*r* = 0.677, *p* = 0.016), whereas the delta value of the TUG test was moderately negatively correlated with the change in stability speed (eyes open *r* = −0.661, *p* = 0.019; Figure 2).

## 4 Discussion

We found that compared to conventional music (administered to the CON group), BBS provides more balance‐related benefits. BBS‐based intervention can significantly improve BI and BDI scores. In the BBS group, the improvement in patients' balance, as measured by the Partial Balance Function Scale, was more closely associated with improvements in physical function. Initially, we hypothesized that patients would improve their balance function by increasing attention or improving cognitive function. However, no changes in any MMSE‐related indicators were detected in either group, which is understandable given that most of our patients already had very high scores, and it is unlikely that significant improvements could be achieved in a short period of 14 days. It must be acknowledged that, while convenient, the MMSE may not be an optimal indicator for assessing attention. Nevertheless, our study showed a greater improvement in BDI scores in the BBS group. Considering the direct correlation between depression and cognitive function, this may also suggest an indirect improvement in various other functions of these patients.

Declining sensory, motor, and spatial abilities due to the loss of proprioception increase the risk of falls in patients with stroke, affecting their independence and leading to depression, further compromising cognitive function, and reducing participation and independence (Daniel et al. 2021; Pollock et al. 2014). In healthy individuals, balance is primarily controlled by the visual and proprioceptive systems. However, peripheral vestibular organs play a more prominent role when information from one of the sensory systems is inadequately transmitted (Peterka 2002). Therefore, vestibular information may additionally assist in maintaining balance in patients with proprioceptive damage, such as patients with stroke. Auditory and vestibular organs are anatomically and functionally interconnected. Auditory organs receive sound stimuli in the form of air‐density waves, which may affect postural regulation (Siedlecka et al. 2015). The auditory system is closely and diffusely connected to the motor system and directly stabilizes it (Koshimori and Thaut 2018). In our study, the BBS group exhibited superior performance in the eye‐open condition during assessments of stability speed and stability area. This finding may be attributed to the increased processing of visual information required in the eye‐open state, which imposes greater attentional demands and necessitates a reallocation of neural resources (Walker et al. 2020). Consequently, the observed improvement in balance during the eye‐open condition may indicate enhanced concentration levels within the BBS group (Wöstmann et al. 2020).

Functional imaging studies have shown that the PFC is associated with auditory and motor responses (Huang and Brosch 2020). Neurons within this cortical region dynamically form functional connections during auditory processing, suggesting that the PFC is involved in the selection of motor responses to both visual and auditory stimuli. Although there is a limited amount of research investigating the effects of auditory stimulation on post‐stroke balance, numerous studies have explored the benefits of visual stimulation, such as virtual reality, in patients with functional impairments. Previous meta‐analyses have indicated that virtual reality can enhance lower limb function, including FMA scores, and improve balance function in stroke patients. These studies underscore the potential influence of both visual and auditory stimuli on motor function. In the current study, following the intervention, the unipodal stance of the BBS group at T1 exhibited significant improvement compared to the CON group. Furthermore, the limits of stability and mobility restriction tests demonstrated improvements from pre‐ to post‐intervention exclusively in the BBS group. The association between lower limb function and balance has been consistently highlighted in the literature (Li et al. 2024). However, in our study, despite the BBS group showing enhancements in stability speed and stability area—indicative of improved balance awareness and motor function‐related indicators such as unipodal stance and mobility restriction tests—there was no significant difference in balance scale scores between the BBS and CON groups at T1.

An increasing number of studies have evaluated the potential rehabilitative effects of music‐based interventions on neurological and non‐neurological diseases (Gonzalez‐Hoelling et al. 2021). Ample evidence suggests that music training and learning promote significant functional and structural changes in the brain (Altenmüller and Schlaug 2015). Moreover, studies have proposed that different regions of the brain respond to different components of music, such as high notes being processed by the temporal lobe, which is also responsible for speech prosody (Patel 2010; Peterson and Thaut 2007), whereas rhythm is processed by the prefrontal motor cortex, cerebellum, and other areas (Berger and Turow 2012). Some studies have suggested that by processing music elements differently, it is possible to access deficient areas and compensate for their functions through alternative transmission paths and connections, as music and neural processing systems are shared. The music therapy can integrate perception and action processes, thereby connecting brain regions that might otherwise not be connected (Leins and Spintge 2008). Cognition and motor function are intricately interconnected (Pang et al. 2018). In our study, as motor function improved, the BBS group exhibited significantly greater enhancements in ADL scores compared to the CON group. Additionally, the BBS group showed significant improvements in BDI scores. These findings may be attributed not only to the interplay between cognitive and emotional processes but also to the enhanced brain connectivity induced by various rhythmic stimulations. This enhanced connectivity may improve the brain's capacity for information integration in regions affected by injury or reduced processing ability (Chen et al. 2016), which condition may contribute to the amelioration of depressive symptoms (Felger et al. 2016), thereby indirectly alleviating depression.

In Lee et al. (2022), BBS intervention also improved anxiety‐ and sleep‐related symptoms, which seemed to be achieved by enhancing arousal memory and emotional control (Lane et al. 1998; Wahbeh et al. 2007). In our study, BBS‐modified music appeared to have better effects than unmodified music. Perhaps unmodified music only stimulated the auditory area, whereas BBS‐modified music activated large‐scale networks throughout the brain. Indeed, the BBS group showed a significant improvement in rhythm perception after medication possibly by stimulating other regions related to music, rhythm, and bilateral auditory stimuli with different intensities. In addition to music, a single auditory stimulus may also affect postural stability. Zhong and Yost examined the effects of auditory spatial cues from a fixed sound source on balance (Zhong and Yost 2013). In their study, postural stability significantly improved in the presence of spatial auditory cues, and further improvement was achieved when combined with visual cues. It has been suggested that a single fixed sound source can provide sufficient spatial cues for the central nervous system to better control postural stability. However, the results of studies on single sound stimuli are not consistent, and unpleasant auditory stimuli may be associated with greater postural sway. The perceived interference scores increased significantly with an increase in frequency and sound pressure level (Chen and Qu 2017). However, it should be noted that although the spatial localization provided by bilateral auditory stimuli is very similar to visual localization, the compensation effect obtained from auditory cues is weaker than that from visual cues (Zhong and Yost 2013). Moreover, music with excessively high frequencies may damage hearing and cause disorders such as hypertension; only specific frequency components do not affect human hearing and can also improve postural stability (Xu et al. 2018). However, the specific components remain unclear.

### 4.1 Limitations

The influence of auditory input on balance, specifically, whether it contributes to increased stability or instability, and the extent to which it plays a role in balance regulation relative to other sensory modalities, cannot be generalized. This necessitates a nuanced examination in specific contexts, considering the intricate interplay of sensory functions. Further research focusing on brain function is essential for a more detailed analysis of the underlying mechanisms.

We did not establish a control group for sham stimulation, which may lead to biased interpretations of the results. We also did not perform functional near‐infrared spectroscopy. Our preliminary experiments demonstrated that the two types of music had consistent effects on the cortex, but BBS could not be completed in the environment necessary for functional magnetic resonance imaging due to objective conditions such as differences in beat and frequency between the two ears (to maintain the different beta, BBS needs to wear headphones, and the metal used in the headphones makes it difficult to enter the regular MRI examination cabin).

Moreover, future improvements in evaluation methods are required. Music therapy may improve function by increasing attention; however, given our focus on balance assessment, we did not perform a dedicated evaluation of attention to substantiate this hypothesis. Moreover, we currently do not know whether, in long‐term therapy, patients become accustomed to or resistant to its effects, resulting in less effective treatment. Future studies should include more protocols to evaluate the effectiveness of BBS or music therapy.

## 5 Conclusion

Compared to conventional music therapy, BBS demonstrates additional benefits for balance function in patients with stroke. Moreover, BBS appears to improve patients’ ADLs and mood. BBS seems to achieve better functional improvement by combining the effects of music and rhythm. However, the specific mechanism remains unclear, and real‐time brain function monitoring is required for further analyses.

## Author Contributions

**Ruijin Chen**: conceptualization, methodology, data curation, writing – original draft. **Lihua Jin**: conceptualization, funding acquisition, writing – original draft, visualization, investigation, validation, formal analysis. **Jin Song**: software. **Juchuan Dong**: investigation, writing – original draft, visualization. **Yongmei Li**: funding acquisition, writing – review and editing.

## Ethics Statement

Ethics approval was obtained by the Second Affiliated Hospital of Kunming Medical University Institutional Review Board (IRB no. PJ‐2023‐189). The trial is registered with the Chinese Clinical Trial Registry (ID: ChiCTR2400088967).

## Consent

Informed consent to treatment was obtained from the guardians of the patient. Prior to participation, written informed consent was obtained from patients or their families.

## Conflicts of Interest

The authors declare no conflicts of interest.

## Peer Review

The peer review history for this article is available at https://publons.com/publon/10.1002/brb3.70671

## Data Availability

The datasets used and/or analyzed for the development of this manuscript are available from the corresponding author on reasonable request.
